# Physical twinning for joint encoding-decoding optimization in computational optics: a review

**DOI:** 10.1038/s41377-025-01810-4

**Published:** 2025-04-15

**Authors:** Liheng Bian, Xinrui Zhan, Rong Yan, Xuyang Chang, Hua Huang, Jun Zhang

**Affiliations:** 1 State Key Laboratory of CNS/ATM & MIIT Key Laboratory of Complex-field Intelligent Sensing, Beijing Institute of Technology, Beijing & Zhuhai, China; 2 Yangtze Delta Region Academy of Beijing Institute of Technology (Jiaxing), Jiaxing, China; 3 School of Artificial Intelligence, Beijing Normal University, Beijing, China

**Keywords:** Optoelectronic devices and components, Imaging and sensing

## Abstract

Computational optics introduces computation into optics and consequently helps overcome traditional optical limitations such as low sensing dimension, low light throughput, low resolution, and so on. The combination of optical encoding and computational decoding offers enhanced imaging and sensing capabilities with diverse applications in biomedicine, astronomy, agriculture, etc. With the great advance of artificial intelligence in the last decade, deep learning has further boosted computational optics with higher precision and efficiency. Recently, there developed an end-to-end joint optimization technique that digitally twins optical encoding to neural network layers, and then facilitates simultaneous optimization with the decoding process. This framework offers effective performance enhancement over conventional techniques. However, the reverse physical twinning from optimized encoding parameters to practical modulation elements faces a serious challenge, due to the discrepant gap in such as bit depth, numerical range, and stability. In this regard, this review explores various optical modulation elements across spatial, phase, and spectral dimensions in the digital twin model for joint encoding-decoding optimization. Our analysis offers constructive guidance for finding the most appropriate modulation element in diverse imaging and sensing tasks concerning various requirements of precision, speed, and robustness. The review may help tackle the above twinning challenge and pave the way for next-generation computational optics.

## Introduction

Computational optics, which integrates optics with computation, stands out as a powerful technique for high-dimensional optical information acquisition^1^. In contrast to traditional optical methods that primarily address the human visual perception of “what you see is what you get", computational optics first employs diverse modulation elements to couple high-dimensional information (such as spatial, spectral, and semantic dimensions) into a low-dimensional optical field that can be directly measured by existing detectors, referred to as the encoding procedure. Then, such techniques employ algorithms to recover high-dimensional information from the measurements, referred to as the decoding procedure^2^. Benefiting from the first-encoding-then-decoding mechanism, computational optics surpass traditional optical limitations of low sensing dimension, low light throughput, low resolution, and so on, developed into a significant and competitive field for optical acquisition and reconstruction^3^. Nowadays, it holds significant value across various fields such as biomedicine, agriculture, and intelligent manufacturing due to its superior and promising performance, even under extreme conditions^4,5^.

**Fig. 1:**
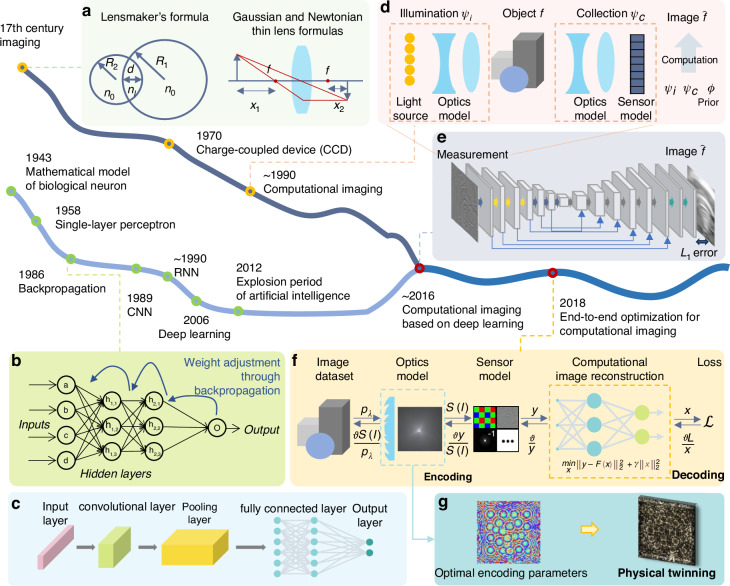
Historical evolution of computational optics, with the horizontal axis representing time and the vertical axis indicating research advances. **a** Illustrates the basic formulations of optics. **b** Presents the concept of backpropagation^6,7^. **c** Depicts the development of Convolutional Neural Networks (CNN)^9–11^. **d** Shows the computational imaging framework^180^. **e** Demonstrates deep learning-based computational reconstruction^181^. **f** Shows the demonstration of end-to-end optimization of both optics and image processing^14^. **g** Demonstrates an exemplar physical twinning process. Adapted with permission^182^. Copyright 2024, Springer Nature

With the great advance of artificial intelligence in the last decade, deep learning has further boosted computational optics with higher precision and efficiency. The deep learning technique dates back to the middle of the 20th century, with theoretical proposals of concepts including backpropagation^6,7^, and single-layer perceptrons^8–11^, as shown in Fig. 1. Over the last decades, rapid advancements in parallel computation and big data processing enabled its practical realizations and propelled the second wave of artificial intelligence^12^. The advancements in artificial intelligence have also led to the emergence of deep learning-based computational optics as a pivotal technique. As depicted in Fig. 1(e), in its framework, the deep learning network functions as the decoding process that inputs the acquired encoded measurement and accomplishes image reconstruction or semantic sensing. This approach overcomes limitations inherent in conventional model-based iterative algorithms, particularly in handling complex tasks such as large-scale imaging^1^ and real-time sensing^13^. Consequently, it has been widely adopted in various applications such as super-resolution imaging^14^, phase imaging^15,16^, and hyperspectral imaging^17^. To further promote efficiency, deep learning-based computational optics focuses on designing high-performance encoding patterns for feature extraction and adopting advanced neural networks for high-quality decoding. However, those methods treat the encoding and decoding processes as separate steps and optimize them independently, which may not be the optimal choice from the perspective of the whole in most cases.

**Fig. 2:**
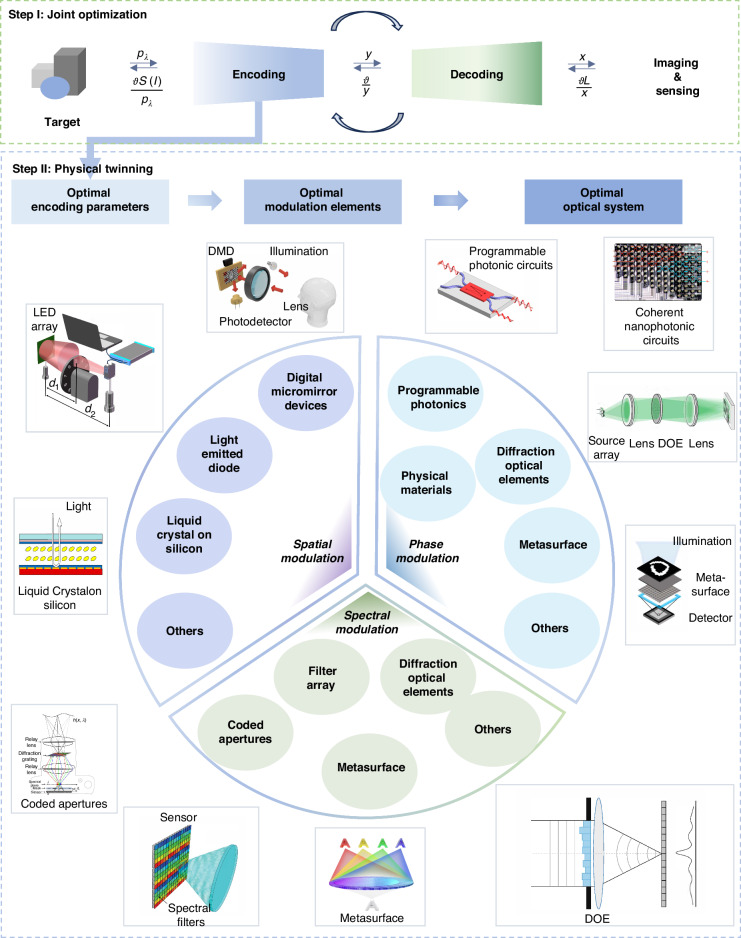
Two steps for joint optimization in computational optics. First, the encoding and decoding processes are optimized in an end-to-end manner, with the encoding treated as network operations using modulation matrices as parameters and decoding managed by a network for target reconstruction or semantic sensing. Second, the optimized encoding parameters are applied to physical modulation elements, and the acquired measurements are fed into the decoding network for subsequent imaging and sensing. The subfigures present exemplar modulation elements, including Deformable Micromirror Device (DMD, adapted with permission^25^. Copyright 2018, Springer Nature), liquid crystal (LC, adapted with permission^183^ under Creative Common CC BY license. Copyright 2018, Huang et al., and LED array (adapted with permission^184^. Copyright 2024, Elsevier) for amplitude modulation; Diffractive Optical Element (DOE, adapted with permission^31^ under Creative Common CC BY license. Copyright 2023, Bernstein et al.), metasurface (adapted with permission^30^ under Creative Common CC BY license. Copyright 2022 Springer Nature), phase-only Spatial Light Modulator (SLM) and others, such as programmable photonic circuit (adapted with permission^29^. Copyright 2020, Springer Nature) and nanophotonic circuit (reprinted with permission^27^. Copyright 2017, Springer Nature) for phase modulation; and filter array (adapted with permission^32^ under Creative Common CC BY license. Copyright 2013, Hagen et al.), DOE (adapted with permission^125^ under Creative Common CC BY license. Copyright 2022, Springer Nature), metasurface (adapted with permission^35^ under Creative Common CC BY license. Copyright 2022, Springer Nature), SLM and others, such as coded aperture (adapted with permission^33^. Copyright 2014, Association for Computing Machinery) for spectral modulation

In most recent studies, an end-to-end optimization method, as illustrated in Fig. 1(f), aims to further enhance the imaging and sensing performance by integrating both encoding and decoding procedures. As shown in Fig. 2, the end-to-end optimization refers to the approaches that utilize an integrated network to optimize both physical encoding and computational decoding parameters. The encoding parameters are then physically twinned in optical systems to achieve high-fidelity imaging and sensing. The system designed by Sitzmann et al. in 2018 is recognized as the first end-to-end optimization demonstration^14,18^. In this framework, a network model maps the targeted optical field directly to the reconstruction result. The encoding procedure, which accounts for optical models including diffractive light propagation, depth, and wavelength-dependent effects, is digitally twinned to neural network layers. The decoding procedure, represented by a neural network, integrates the encoding procedure within its network layers. Through iterative training of the encoding-decoding network, the decoding loss function provides feedback for simultaneously optimizing the encoding parameters. The optimized encoding parameters assist in improving the decoding results, thereby leading to a notable efficiency enhancement. The end-to-end optimization strategy demonstrates effective improvement on encoding efficiency and decoding precision and has been favored in real-time, high-precision imaging and sensing^13,19^.

With the optimized encoding parameters, there follows a subsequent reverse physical implementation in the end-to-end optimization framework. It requires modulation elements to physically realize the optimized encoding parameters, which we refer to as the “physical twinning" procedure that denotes the mapping process from mathematical design to physical implementation (as shown in Fig. 1(g)). However, the end-to-end optimized encoding-decoding framework struggles to account for all physical restrictions, including bit depth, numerical range, stability, *etc*. Recent studies have attempted to tackle these challenges. First, to deal with the bit depth gap, mathematical binarization algorithms^20,21^, such as dithering, and binary encoding network operations^22^, can be applied to translate encoding parameters onto precision-limited devices. For the challenge of numerical range, where optimized modulation matrices contain negative or excessively large values, these matrices can be decomposed into multiple non-negative sub-matrixes^21^. Physical twinning is then realized by applying temporal weighting and accumulating measurements from each sub-matrix. Third, environmental factors such as temperature^23^ and service life, affect the stability of modulation elements and should also be incorporated as part of the encoder’s optimized parameters. However, accounting for all these variables in the optimization process would lead to a highly complex and unsystematic structure. Therefore, despite the existing advancements and auxiliary strategies for physical twinning, it remains challenging to balance high-precision results with suitable modulation elements of various experimental requirements.

In this regard, we delve into the physical twinning process in end-to-end jointly-optimized computational optics. As illustrated in Fig. 2, the following sections present common modulation elements across amplitude^24–26^, phase^27–31^, and spectral^32–35^ modulation dimensions. In each section, we provide a list of representative modulation elements suitable for joint optimization, along with introductions to their principles, applications, and limitations. This work offers constructive guidance for finding the most appropriate modulation element in diverse tasks concerning various requirements of precision, speed, and robustness, and helps to promote the development of jointly-optimized computational optics.

## Amplitude modulation

Amplitude refers to the maximum deviation of light’s electromagnetic field from its average value during wave propagation. The amplitude modulation, also known as intensity modulation, is typically a two-dimensional spatial function that alters light intensity in a controlled manner. This kind of modulation can be achieved in either transmissive or reflective modes, where the light amplitude is controlled by adjusting the local transmission or reflection coefficients of the optical medium^36^. A common form is binary amplitude modulation, where the amplitude is adjusted between two distinct reflection or transmission values.

Through the last several decades, computational optics systems utilizing amplitude modulation have been widely applied across various fields, such as multi-scale gigapixel photography^37^, single-pixel imaging^25^, wavefront shaping^38^, imaging through turbid media^39^, ultrasound high-speed imaging^40^, and so on. Multiscale gigapixel photography^37^ aims to establish a camera array for scalable gigapixel imaging. Single-pixel imaging, also known as computational ghost imaging, maximizes spatial redundancy by spatial encoding and coupling^41^. It applies a sequence of spatial modulation patterns and uses a single-pixel detector to record the cumulative light intensity^19,22^. Other systems based on amplitude modulation, such as wavefront shaping that spatially modulates incident light to focus or penetrate scattering media^38^, also adopt a similar imaging model. In these systems, amplitude modulation can be mathematically represented as a Hadamard product during the encoding process, while the modulated light is fed into the decoding algorithms to achieve specific goals.

Adler et al. introduced end-to-end joint optimization into amplitude modulation for compressed imaging^13,42^. When applied to the MNIST dataset^43^, their method achieved an impressive 6.46% classification error, significantly outperforming the state-of-the-art error rate of 41.06% at a sampling ratio of 0.1. Subsequent studies have further explored and validated joint optimization in amplitude modulation, leading to wide applications in real-time imaging^44^, object tracking^45^, and terahertz imaging^46^.

**Fig. 3:**
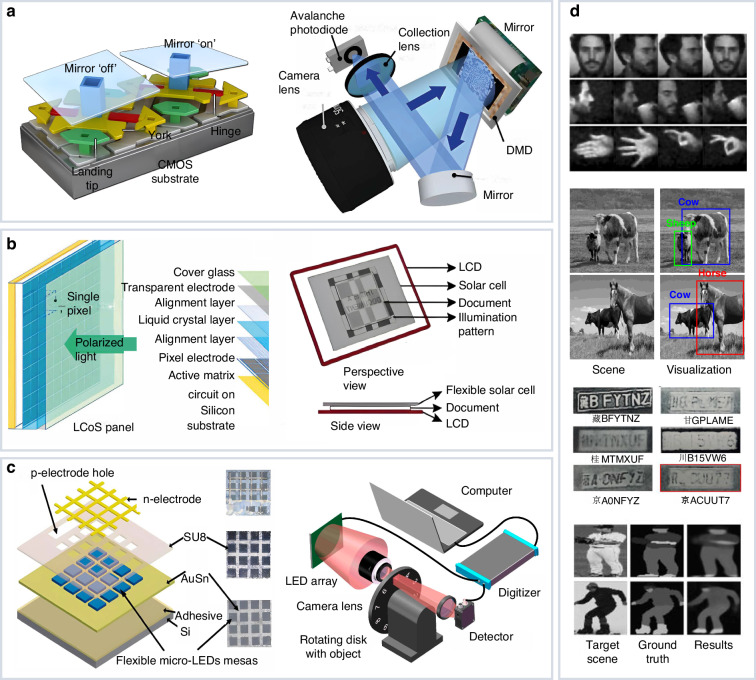
Amplitude modulation elements for end-to-end joint optimization applications. **a**–**c** Depict three typical devices including DMD (the left image is adapted with permission^185^. Copyright 2017, Springer Nature. The right image is reprinted with permission^162^ under Creative Common CC BY license. Copyright 2017, Phillips et al.), LC (the left image is adapted with permission^186^ under Creative Common CC BY license. Copyright 2019, Li et al. The right image is reprinted with permission^63^ under Creative Common CC BY license). Copyright 2019, Optical Society of America, and LED (the left image is reprinted with permission^187^ under Creative Common CC BY license. Copyright 2016, Optica Publishing Group. The right image is reprinted with permission^26^ under Creative Common CC BY license. Copyright 2022, Huang et al). The left panel of each subfigure shows the schematic design of each device, and the right panel shows its experiment setup. **d** Depicts corresponding representative applications, including imaging (adapted with permission^44^ under Creative Common CC BY license. Copyright 2018, Springer Nature), target detection (adapted with permission^57^. Copyright 2023, Optica Publishing Group), multiple target recognition (reprinted with permission^19^. Copyright 2022, Optica Publishing Group), and object segmentation (reprinted with permission^56^. Copyright 2023, Elsevier)

Amplitude modulation is mainly implemented using Spatial Light Modulators (SLMs), which can spatially manipulate electromagnetic waves. In the following of this section, we introduce three widely used SLMs for amplitude modulation, including Deformable Micromirror Device (DMD), Liquid Crystal (LC) devices, and Light Emitting Diode (LED), as shown in Fig. 3. Overall, DMD is the most popular amplitude modulation element due to its high resolution and high-speed modulation capability, though the high-speed modulation is limited to the binary mode only. LC offers a low-cost amplitude modulation solution, but it is insufficient in modulation speed and sensitive to temperature conditions. LED takes advantage of high-speed modulation and color performance. However, its spatial resolution is relatively lower than the others.

### Deformable micromirror device (DMD)

DMD was early developed by Hornbeck in the 1980s^47^, starting as a 128 × 128 array addressed by an underlying array of MOS transistors. Each micromirror in the array can be individually rotated by +12 degrees or −12 degrees, representing on and off states for light transmission, respectively. At present, DMD is one of the most popular SLMs for amplitude modulation, mainly due to its high resolution (1920 × 1080 pixels or higher), high refresh rate for binary patterns (22 kHz or higher). Consequently, DMD-based computational optical systems benefit from the high-speed modulation for reliable detection at even invisible wavelengths such as near-infrared^48^ range and mid-infrared^49^ range.

DMD-based computational imaging setups are widely adopted in various experimental end-to-end joint optimization systems, with convolutional neural networks^46,50^, recurrent neural networks^51^, etc. These systems have demonstrated wide applications including real-time single-pixel imaging^44,52^, super-resolution imaging^53^, Light Detection and Ranging (LiDAR)^50^, occluded target imaging^54^, imaging through scattering media^55^, terahertz imaging^46^, and so on. To enhance efficiency for semantic sensing tasks, image-free sensing techniques bypass the complex image reconstruction process and extract semantic information directly from compressed measurements^19,22,56–60^, offering efficient and lightweight sensing solutions. Their physical twinning process using DMD has successfully demonstrated multiple applications, including character classification^22,58,59^, multi-character recognition^19^, segmentation^56^, detection^57^, object tracking^45^, semi-supervised sensing^61^, few-shot sensing^62^, optical encryption^58^, optical neural network^60^, and so on. In these experiment systems, end-to-end optimization shows significant improvement on decoding performance. For instance, it enhanced Chinese characters’ perception accuracy by up to 24% compared to conventional methods^55^.

Despite the high-speed modulation and effectiveness at invisible wavelengths, DMD has certain limitations. First, each micromirror on DMD is fixed to either an “on" or “off" position, and therefore provides high-speed modulation only for binarized patterns. For grayscale modulation patterns with multiple bits, it needs to be implemented multiple times at an exponential scale. Each grayscale pixel’s intensity is temporally weighted within a predefined time frame. Such a process largely degrades modulation efficiency. For example, a common DMD can only generate 8-bit grayscale patterns at a frame rate of around 250 Hz^20^. To address this limitation, the pattern binarization strategy has been introduced to approximate grayscale modulation matrices into binary ones^20^. However, the pattern binarization process disrupts the optimal matching between physical encoding and computational decoding, and consequently results in information loss and deviates from global optimum. Fu et al.^22^ report a two-step training strategy for joint optimization. In the first step, an end-to-end network is trained with a global scaling factor *α* relating the optimal modulation pattern values and their corresponding binary ones. In each backward propagation, the grayscale weights are updated using the gradients, and the optimal binary weights can be obtained at the end of the first training stage. In the second step, the encoding parameters are fixed as binary values, and the decoding network is fine-tuned to better match the fixed binarized encoder and improve sensing performance. This two-step training strategy can effectively enhance optical performance with high-speed binary modulation. Another limitation of DMD is that it requires a fine-tuned lens-based imaging system to project structured light patterns. This requirement is easily corrupted by aberrations and poses challenges on establishing compact imaging systems^63^.

### Liquid crystal (LC)

Liquid crystal (LC) materials are organic compounds that exhibit both liquid and crystalline properties. They are characterized by anisotropic dielectric constants and refractive indices. By leveraging these anisotropic properties, the orientation of liquid crystal molecules can be controlled via an electric field, allowing for the modulation of light propagation direction. This principle underlies the development of Liquid Crystal Display (LCD), which utilizes the birefringence of liquid crystal molecules. As a widely used SLM, LCD achieves grayscale control by applying different voltages to pixel units in conjunction with orthogonal polarizers. Over time, advancements led to the development of the Liquid Crystal on Silicone (LCoS) technique, which combines silicon-based integrated circuits to offer a higher spatial resolution (up to million pixels), better light energy utilization (70–80% light throughput efficiency, and 2000 lumens/cm^2^ luminous densities^64^), and faster pixel switching speed (response time of ~ 1 ms at gray level^64^) compared to LCD^65^. LC-based modulation elements maintain certain advantages over DMD, including the ability to project grayscale patterns, the applicability of phase modulation, and the elimination of lens-related degradation such as optical distortion, aberrations, and chromatic dispersion^63^.

LCD and LCoS have been widely employed for amplitude modulation benefiting from their advantages in high-precision modulation and constructing lensless imaging systems. For instance, Zhang et al. adopted LCD to construct a compact lensless single-pixel imaging system in which the compact scanner is as thin as 2.48 mm, and achieved grayscale and true-color scanning as well as accurate optical character recognition^63^. LCoS has been widely applied in Structured Illumination Microscopy (SIM), including 2D SIM^66^ and 3D SIM^67^, offering high frame rates with moderate cost. For instance, Lu-Walther et al. designed a FLCoS-based two-beam SIM fluorescence microscope and realized a lateral resolution of ~100 nm at a maximum raw data acquisition rate of 162 frames per second (fps) with a region of interest (ROI) of 16.5 × 16.5 μm^2^^68^. Hannebelle et al. designed a SIM system that places LCoS at the primary image plane of microscope^69^. This system, in combination with a polarizing beam splitter (PBS) in the amplitude mode, serves a computer-controlled modulation function that reduces demands for precise positioning and alignment of multiple optical elements. The striped illumination pattern of SIM^70^ can also be jointly optimized under certain constraints in the encoder module. Therefore, although these LCoS-based imaging systems do not currently utilize end-to-end joint optimization, their flexible design makes them promising for jointly-optimized physical twinning.

LC-based amplitude modulation elements have certain limitations. First, constrained by the intrinsic properties of LC, these elements are sensitive to temperature variations, leading to performance degradation at extremely high and low temperatures. For instance, the operating temperature of the common Thorlabs WUXGA LCoS is restricted between 10 ^∘^C and 40 ^∘^C^23^. Second, the modulation speed of LC-based elements still lags behind that of DMD, for around 100 Hz^63^. Ferroelectric Liquid Crystals on Silicon (FLCOS) maintain a faster modulation speed of up to several kHz, more than an order of magnitude higher than nematic liquid crystals^71^, which is however relatively more complex to fabricate and may suffer from significant degradation over long-term use or frequent switching^72,73^.

### Light emitted diode (LED)

The LED array consists of a two-dimensional diode matrix where cathodes are connected by rows and anodes by columns. By controlling the electrical current through each diode to vary the intensity of the emitted light, it enables precise modulation of light’s amplitude. Within an LED array, the individual current of each diode in each column or row can be independently controlled, facilitating end-to-end optimized modulation patterns for physical twinning. Moreover, LED can achieve remarkably high frequencies for amplitude modulation, with modulation speed up to around 50MHz^74^.

LED has diverse applications in computational imaging as a structured light encoding device. For instance, Xu et al. report a computational ghost imaging scheme utilizing an LED-based high-speed illumination module. This approach achieved a frame rate of 1000 fps with a resolution of 32 × 32 pixels^75^. Salvador et al. used a low-cost color LED array for structured illumination, enabling a high refresh rate of up to 10 kHz. This system can be further extended for 3D imaging by incorporating additional photodiodes placed at different positions^76^. Although the above-mentioned publications have not realized physical twinning of end-to-end optimization, the flexible design of LED offers the potential to realize learning-based encoding patterns.

LED faces a serious limitation of low spatial resolution. The size of a typical LED array with a resolution of 32 × 32 pixels is around 64 mm × 64 mm^75^. The limited spatial modulation precision limits the system’s ability to produce fine modulation patterns. To achieve a higher spatial resolution, it is necessary to construct an expanded LED array. However, an extended LED array is too large in size for compact imaging systems. Moreover, it becomes challenging for a single FPGA to provide enough I/O ports for a large-scale LED array^75^, leading to a more complex system with a slower response speed. This increase in complexity can complicate the setup and potentially reduce the efficiency and practicality of the system for certain applications.

### Others

In addition to the above common SLMs, there are alternative elements for amplitude modulation. For example, Hahamovich et al. implemented cyclic patterns using a spinning mask, achieving rapid amplitude modulation of up to 2.4 MHz, surpassing the speed limitation of configurable SLMs and enabling real-time visualization of dynamic objects^77^. However, this technique requires a fixed design of cyclic patterns, making it not feasible for controllable physical twinning in end-to-end joint optimization. There are also several other materials with potential for joint optimization. For example, He et al. realized single-pixel neutron imaging using Hadamard masks made of gadolinium oxide (*Gd*_2_*O*_3_), which had a considerable resonance and absorption cross section for thermal neutrons^78^.

## Phase modulation

Both amplitude and phase are fundamental dimensions for describing the wave characteristics of light^79^. Phase represents the propagation delay caused by the interaction between incident wavefront and matter. Phase modulation alters the spatial phase distribution of a wavefront, thereby influencing light convergence, propagation, and delay to achieve specific objectives. The phase modulation-based encoding layer typically involves a Hadamard product between the incident wavefront and the phase pattern. In experimental implementation, this process occurs automatically as the light propagates through the patterns. Compared to amplitude modulation, phase modulation preserves a higher ratio of light intensity.

**Fig. 4:**
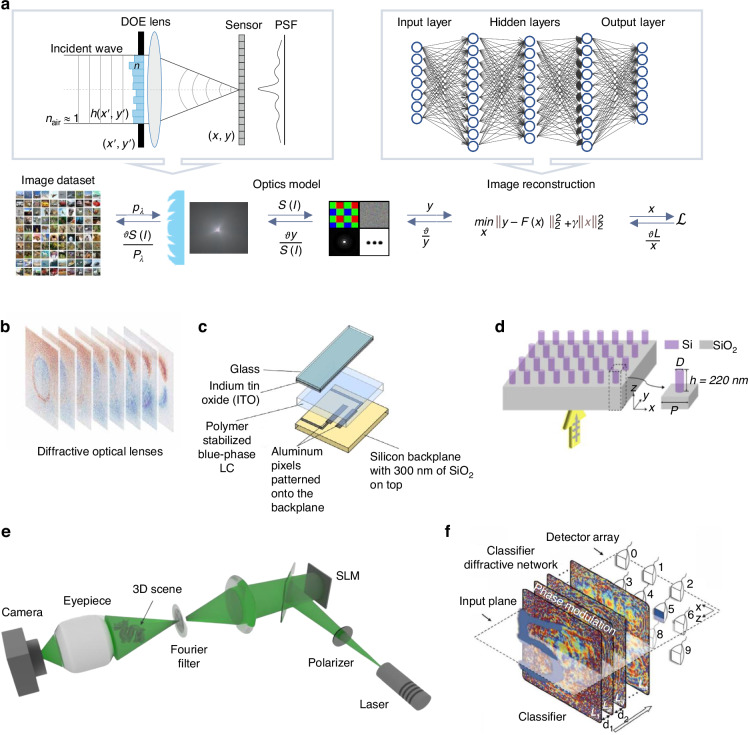
Phase modulation elements for end-to-end joint optimization applications. **a** Illustrates the framework of joint optimization. Adapted with permission^125^ under Creative Common CC BY license. Copyright 2022, Springer Nature. **b**–**d** Present examples of the structures of DOE (adapted with permission^188^ under Creative Common CC BY license. Copyright 2024, Springer Nature), phase-only SLM (reprinted with permission^189^. Copyright 2014, Optical Society of America), and metasurface (adapted with permission^190^ under Creative Common CC BY license. Copyright 2019, Optica Publishing Group), respectively. **e**, **f** Depict two exemplar experiment setups of end-to-end joint optimization applications in the phase dimension, showcasing phase imaging (adapted with permission^93^. Copyright 2011, Springer Nature) and D^2^NN (reprinted with permission^85^. Copyright 2018, Lin et al.)

Phase modulation has been widely applied in computational optics. In quantitative phase imaging^80–82^, phase modulation is able to extend imaging throughput, field of view, and imaging resolution. Adaptive optics^83^ employs phase modulation to correct wavefront distortions caused by atmospheric turbulence or optical aberrations, significantly enhancing image resolution in astronomy and microscopy. Wavefront shaping^84^ uses phase modulation to control light propagation through complex media, enabling deep-tissue imaging. Recent years have seen a surge in end-to-end optimization of the phase modulation patterns and imaging or sensing processes^14^. A representative example is diffractive deep neural Networks (D^2^NN)^85^, as shown in Fig. 4(f). D^2^NN consists of several diffraction layers that represent optical operation, each of which is composed of hyperparameters that represent physical light-transferring procedures. The implementation of D^2^NN usually requires two steps. First, in the training stage, a task-based D^2^NN is built and trained until convergence, with the trainable hyperparameters denoting phase modulation patterns. In the second step, the inference stage, the reconstructed images or sensing results can be directly visualized at the detector when light propagates through these phase patterns. This method enables D^2^NN to achieve high-precision inference^86,87^ at a speed of light propagation without external power, potentially overcoming the limitations of high energy consumption and low efficiency in electronic computing.

The above-mentioned studies were based on various phase modulation elements. As shown in Fig. 4, typical phase modulators include Diffraction Optical Element (DOE), phase-only Spatial Light Modulator (SLM), and metasurface. Each type of modulator has its own advantages and disadvantages. DOE provides low-cost and plug-and-play phase modulation, but it is usually bulky and cumbersome. Phase-only SLM offers high spatial resolution and programmable modulation using liquid crystal, but it is relatively expensive and bulky and has low optical efficiency. Metasurface is compact and lightweight, allowing for easy integration, but it is difficult to fabricate and involves high design costs.

### Diffraction optical element (DOE)

Diffraction Optical Element (DOE) manipulates the phase of light by introducing optical path differences on grating’s structure. These elements can precisely control the diffraction angles, which affect the direction and distribution of the diffracted light. Designing DOEs involves prior calculations to determine the optimal arrangement and dimensions of the microstructures. The techniques such as rigorous coupled-wave analysis^88^ and finite-difference time-domain (FDTD) simulation^89^ are commonly employed to model and optimize DOE performance. The fabrication of DOEs typically utilizes high-precision lithography or 3D printing techniques to pattern microscopic structures on silicon or glass substrates^90^.

DOEs have been employed in numerous applications such as beam shapers, beam splitters, and diffusers^91^. Due to their low cost (as low as a few dozen dollars) and plug-and-play convenience, DOEs have naturally become a common choice for physical twinning in end-to-end joint optimization. A notable study by Li et al. introduced a joint optimization approach that optimizes both the DOE placed in front of the projector lens and the compensation network used for image deblurring^92^. This technique can extend the projector’s Depth-of-Field (DOF) by a minor modification to the projector. The compensation network demonstrated significant advancement in radiometric compensation, particularly in terms of computational efficiency and image quality. Shi et al. demonstrated optimized 3D printing for fabricating diffractive layers, showing great performance of real-time 3D holography with low memory requirement (below 620 KB), high frame rate (60 Hz), and high resolution (1920 × 1080 pixels) on a single consumer-grade graphics processing unit^93^. To address the discrepancies between optimized digital modulation patterns and the actual fabricated ones resulting from manufacturing imperfections and model approximations, decoding networks were typically fine-tuned through several iterations using the real calibrated patterns.

Although DOE is highly effective for phase modulation, it has several inherent limitations that impede its broader applications. First, the fixed structural configuration limits DOE’s adaptability in dynamic optical systems^94^. This rigidity means that once a DOE is fabricated, its phase modulation properties cannot be altered without creating an entirely new element, making real-time adjustment impossible. Second, DOE usually suffers from a low diffraction efficiency (40% to 70% for binary DOEs)^95^, where not most incident light is effectively modulated as desired. This results in low light efficiency and unwanted scattering or diffraction orders, which degrade the performance of optical systems. Third, most DOEs are bulky and cumbersome^91^, posing an integration challenge for compact and portable devices.

### Phase-only spatial light modulator (SLM)

Phase-only SLM modulates the phase of light in a pixel-wise manner, typically using liquid crystal^96^ or microelectromechanical systems (MEMS)^97^. These devices can independently adjust the optical properties of each pixel in response to electronic signals, which alter the refractive index (in liquid crystal SLM) or the physical displacement (in MEMS SLM) to achieve desired phase modulation.

SLM is crucial for applications that require dynamic manipulation of phase, including both conventional optical imaging and end-to-end joint-optimized computational imaging^98^. In terms of conventional optical imaging, SLM can dynamically generate and project high-quality holograms to realize holographic imaging^99^. In adaptive optics, SLM is used to correct wavefront distortions in real time, improving image resolution in astronomical observations and microscopy^100^. Besides, SLM also plays a crucial role in beam shaping^101^, where it is used to tailor the intensity profile of laser beams. In terms of end-to-end joint optimized computational imaging, Chen et al. report a large-scale, cost-effective, complex-valued, and reconfigurable diffractive all-optical neural network system in the visible range based on cascaded transmissive twisted nematic SLMs with a distinct statistical adversarial property^102^. Wang et al. used SLM to implement D^2^NN and realized orbital angular momentum spectrum measurement^103^. Pinilla et al. jointly optimized the phase patterns in front of an image sensor and realized achromatic extended-depth-of-field imaging^104^. Compared to conventional lens-based imaging systems, they achieved around 2 dB improvement on PSNR. Due to its high accuracy and programmable phase modulation capabilities, the SLM-based encoding layer can accurately implement digital patterns with minimal mismatch caused by fabrication errors.

Despite the dynamic and pixel-wise modulation capabilities, SLM has several limitations. First, high-performance and high-resolution SLM is usually expensive at tens of thousands of dollars^105^. Second, SLM also suffers from low diffraction efficiency (60% to 90%) due to light loss at the liquid crystal layers or MEMS mirrors^106^. Third, the calibration of SLM is generally complex and time-consuming^107^.

### Metasurface

Metasurface is artificially designed and manufactured with special physical properties, such as negative refractive index, negative magnetic permeability, and negative dielectric constant^108^. By altering the basic unit structure, shape, orientation, arrangement and properties, metasurface can effectively manipulate various dimensions of light waves. In terms of phase modulation, metasurface introduces phase shifts through the interaction of light with the meta-atoms. Each meta-atom can be designed to impart a specific phase delay, which cumulatively shapes the wavefront of the transmitted or reflected light. Benefiting from the advantages of lightweight, easy integration, and high light efficiency^109^, metasurface is particularly suitable for physical twinning of phase modulation in end-to-end joint optimization.

Metasurface has been widely applied in various optical imaging/sensing techniques^15,110^. In conventional optical imaging, Zheng et al. developed metasurfaces for generating high-resolution, high-efficiency holographic images, achieving a light efficiency of 80%^110^. Subsequently, various metasurface-based holography techniques have demonstrated great success^111–113^. For joint optimization, Ghorbani et al. explored the use of metasurface to construct D^2^NN, which can perform on-chip multi-channel sensing in the visible range^30^. Tseng et al. developed a fully differentiable learning framework that learns a physical metasurface structure in conjunction with a neural feature-based image reconstruction algorithm, which achieved an order of magnitude lower reconstruction error than existing approaches^114^. While the above-mentioned passive-structure-based D^2^NN platforms can perform complex functions based on computer-aided neural network design, they still rely on computers to optimize parameters and require prior environment information. Liu et al. report a programmable metasurface where each meta-atom on the metasurface was integrated with two amplifier chips, acting as an active artificial neuron. This architecture can handle various deep learning tasks including image classification, mobile communication encoding/decoding, and real-time multi-beam focusing^115^. Similar to DOE-based techniques, the metasurface-based phase modulation encoding process also suffers from mismatches between optimized digital patterns and actual fabricated ones, which would degrade the performance of decoding network. To address this issue, most studies typically refine the decoding network parameters using the real calibrated patterns.

Despite the above advantages, metasurface faces several limitations for phase modulation. First, the fabrication process is complex and costly, requiring high manufacturing precision for designed microscopic structures^116^. Second, while metasurface offers excellent control over multiple optical properties, it suffers from efficiency loss due to imperfect nanostructure fabrication and alignment^117^. Third, the scalability of metasurface is limited by the difficulty in maintaining uniformity across large areas^118^. Further studies of physical twinning and low-cost fabrication techniques continue to be crucial for advancing the practical applications of these advanced optical materials.

### Others

Besides the above-mentioned DOE, SLM and metasurface, there are also some other devices available for phase modulation. For example, scattering/holography diffusers, which are widely used for phase modulation and can achieve super-resolution and large depth-of-field tasks, are seldom considered for optimization^80,119^. Semiconductor optical phased arrays (OPA) offer a fast modulation speed of up to 10 GHz, but their numerical randomness makes reverse design challenging^28^. DMD can be employed for phase modulation with specially designed optical circuits^120,121^. However, DMDs are essentially binary modulators, and their technological maturity and diffraction efficiency are often insufficient for multiple-phase modulation applications^122^. Nanophotonic circuits based on optoelectronic devices offer programmability but are hindered by a large footprint, posing challenges for the realization of large-scale neural networks^27^. While the aforementioned novel devices can realize phase modulation, their complex structures, randomness, and low diffraction efficiency and resolution hinder end-to-end joint optimization. Thus, it is essential to comprehensively evaluate the use and performance of these devices when selecting a phase modulator.

## Spectral modulation

Dating back to the 17th century, spectrum was defined to represent the light intensity of a range of wavelengths^123^, which reveals the intrinsic spectral frequency of an electromagnetic wave. Spectral modulation denotes selecting and encoding the intensity of a range of wavelengths, namely a function of wavelength. This modulation relies on manipulating different optical parameters in the propagation path, such as the reflection index or transmittance^124^. When the light goes through the medium, the intensity or phase of each wavelength will change differently, leading to a change in spectrum. Generally, there are three common strategies for spectral modulation, including directly using specific materials for filtering, indirectly using diffraction to map spectral information into phase for modulation, and indirectly using refraction to map spectral information into space for amplitude modulation^125^.

**Fig. 5:**
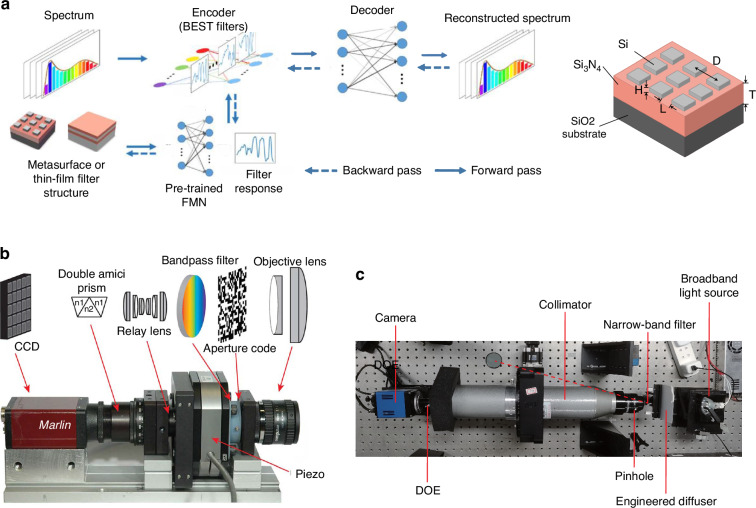
Spectral modulation elements for end-to-end joint optimization applications. **a** Illustrates the framework of and fabrication for the joint optimization in the spectral dimension. Adapted with permission^137^. Copyright 2021, John Wiley & Sons. **b** and **c** show several representative spectral imaging systems, in which **b** presents the Coded Aperture Snapshot Spectral Imager (CASSI, image reprinted with permission^191^. Copyright 2010, Optical Society of America), and **c** shows the DOE-based snapshot hyperspectral imaging system (reprinted with permission^192^ under Creative Common CC BY license. Copyright 2023, Optica Publishing Group)

The development of spectral modulation advances computational spectral imaging by selective manipulation and precise encoding of different wavelengths of light. Different from the conventional one-dimensional or two-dimensional scanning-based spectral imaging techniques^32,126^, computational spectral imaging allows for the acquisition of two-dimensional modulated projection data of the target spatial and spectral information with a single imaging exposure^32,125^. Through subsequent data decoupling and processing, the three-dimensional spectral data cube can be reconstructed^125^. In recent years, there has been a surge in end-to-end optimization of spectral modulation in computational imaging, as shown in Fig. 5. Similar to amplitude and phase modulation, spectral modulation in the encoding layer is represented by a Hadamard operation, with each spectral component integrated through multiple network channels. Considering the physics-based propagation of light and its interaction with optical elements or devices, end-to-end optimization utilizes advanced algorithms to enhance imaging resolution and accuracy^127^. It holds applications in diverse fields such as astronomical observation, remote sensing, agriculture, and biomedicine^128,129^.

Conventional spectral modulation elements such as prism and diffraction grating are widely used due to their simplicity. However, they may be constrained by size, diffraction limit, and reliance on light propagation^130^ in practical setups. In recent decades, there have developed multiple devices for spectral modulation, including the following four representative ones of filter array, Diffraction Optical Element (DOE), Spatial Light Modulator (SLM) and metasurface, as shown in Fig.5(b-d). Each one has its respective pros and cons. Filter array offers precise spectral selectivity but maintains spectral crosstalk and limited flexibility^131^. DOE can improve light throughput and reduce system volume^125^, but it requires a sophisticated fabrication process and is sensitive to environmental fluctuations. SLM offers high spatial resolution and fast response, but it may suffer from limited light throughput and requires complex drive electronics^125^. Metasurface has versatility and compactness (tens of nm to microns^130^), but it requires precise and costly nanofabrication.

### Filter array

Filter array (also known as multispectral filter array) consists of multiple filters arranged in a spatial pattern, and each filter is designed to selectively transmit specific wavelengths of light. By carefully utilizing materials of different optical properties to design arrays, filter array enables precise control over light spectrum at each location. These filters can be designed to allow only a narrow band of wavelengths (narrowband filters) to pass with FWHM being as low as ~2 nm^132,133^, or they can transmit a wide range of wavelengths (broadband filters) or block a certain range (band-stop filters, also known as notch filters). Upon different requirements, they can achieve precise spectral selection and are relatively easy to integrate into different optical devices and systems^131^. Additionally, the variety of manufacturing materials, ranging from traditional materials to new ones such as dielectric coatings, glass, or polymers, could achieve diverse results for enhancing or suppressing spectral components.

Benefiting from its spectral selectivity and easy integration advantages, filter array has been extensively applied in computational optics. Studies mainly focused on optimizing the pattern of filter arrays using deep learning methods^134^. For instance, Zhang et al. denoted the transformation matrix of filter array using a encoding neural network, which was learned simultaneously with the decoding network^135^. To realize physical twinning, the network was designed to learn non-negative and smooth spatial-spectral patterns. Based on the joint optimization framework, the results improved PSNR for more than 1.7 dB compared to traditional methods. In recent years, broadband filters have attracted much attention for hyperspectral imaging due to their high throughput. Zhang et al. designed a broadband-coded hyperspectral camera based on deep learning and achieved a processing speed increase of 7000–11,000 times and approximately a 10–fold improvement on noise tolerance^136^. Using 16 broadband filters, the camera realized multispectral imaging of 480 × 640 pixels at 1 nm spectral resolution in the range of 400–700 nm. Song et al. reported a spectral encoder and decoder network for joint optimization of filters. Compared to conventional non-learning methods, the designed filter array realized 30 times enhancement of reconstruction accuracy and better tolerance on fabrication errors in the spectral range of 400–700 nm^137,138^.

Despite its versatility, filter array has certain limitations. One constraint is their finite bandwidth and spectral crosstalk. End-to-end optimization has demonstrated its ability to alleviate this issue^135^. Besides, an existing filter array has fixed spectral characteristics, limiting its adaptability to changing experimental requirements or spectral ranges.

### DOE

DOE operates on the diffraction principle to distinguish different wavelengths as light propagates. It can realize complex optical functionalities^139^ with high throughput, enabling high-precision spectral modulation with spectral peak location accuracy better than 1 nm and peak separation resolution of 3 nm^140^). Additionally, DOE’s compact design (~1 μm thickness^141^) allows integration into miniaturized optical systems, making them suitable for portable and space-constrained applications.

In recent years, DOE has been widely applied for spectral imaging. The common DOE-based spectral imaging framework builds on the DOE-Fresnel diffraction or DOE-Lens system^125^. For instance, Lv et al. built an Aperture Diffraction Imaging Spectrometer (ADIS), which employs diffraction-based spatial-spectral projection for encoding. It improved the diffraction efficiency to 75% and achieved sub-super-pixel spatial resolution (1056 × 1536 pixels) in the spectral range of 453–646 nm^142^. Mengu et al. reported a diffractive optical network-based multispectral imaging system, which adopted 3D-printed diffractive surfaces. Considering the layer-to-layer misalignments, they were modeled as random variables and incorporated into the forward training model. In this way, the deep learning-based evolution of the diffractive surfaces is enforced to converge to solutions that show resilience against implementation errors. The joint optimized system achieved 16-channel snapshot multispectral imaging in the range of 450–700 nm with > 79% transmission efficiency^143^. Baek et al. reported a DOE-based spectral-depth imaging system, which was jointly optimized by a differentiable simulator and a neural network-based reconstruction method. Via automatic differentiation, the end-to-end network optimizes DOE’s PSF at different wavelengths and depths, which can simultaneously couple spectral and depth information into a snapshot. To avoid additional complexity of employing physical fabrication constraints, they optimized the unwrapped phase instead of DOE height. Upon a single shot, the technique can simultaneously realize multispectral imaging with 3840 × 5760 pixels in the range of 420–660 nm and depth imaging at 0.4–2 m^34^.

While DOE offers multiple advantages, its fabrication requires complex and precise microfabrication. DOE’s performance is sensitive to fabrication imperfections, which limits its scalability and increases production costs. Besides, DOE’s efficiency is also affected by diffraction efficiency loss (40–70% for binary DOE^95^), which means that a large portion of incident light is not properly guided to the intended diffraction order. In addition, factors such as incident angle and mechanical stress can also affect DOE’s stability and performance. These challenges require careful consideration in the processes of optical design, fabrication, and application.

### SLM-based coded aperture

In a coded-aperture spectral modulation system, the spectrum is usually dispersed into the spatial domain by a prism or optical grating. Then, a coded aperture is placed to selectively manipulate the dispersed spectrum. One of the most common and flexible devices to achieve a coded aperture is SLM^144^, which can block or unblock target light to modulate the spatial distribution of different wavelengths. This modulation allows precise adjustment and selection of specific wavelengths, thus increasing the flexibility and accuracy of spectral imaging. The key advantages of SLM lie in its high resolution, fast response, and programmability.

Benefiting from SLM’s flexibility, Li et al. reported a non-mechanical spatio-spectral modulation method that realized single-pixel multispectral imaging with 128 × 128 pixels in the 420–720 nm range at 2 Hz^145^. In recent studies, the employment of SLM in Coded Aperture Snapshot Spectral Imager (CASSI)^146^ has gained much attention. CASSI encodes the hyperspectral datacube into a single compressed measurement and then employs algorithms to reconstruct the underlying hyperspectral information. In the past five years, a series of end-to-end optimization algorithms have emerged to maximize the information content of compressive measurements. Wang et al. utilized a convolutional neural network to jointly optimize the coded aperture and subsequent reconstruction algorithm. By improving the spectral and spatial correlations, the reconstruction accuracy was improved by about 2 dB compared with other methods^147^. Shi et al. reported a self-adaptive encoding method for multispectral imaging, which uses SLM’s flexibility to adaptively optimize coded patterns^144^. In addition, to ensure flexible physical twinning, they designed an element-wise binarization function to transform the optimized continuous encoding matrix into the binary mask form. The adaptive strategy improves hyperspectral reconstruction accuracy by at least 0.8 dB.

SLM has several limitations for spectral modulation. One is its limited diffraction efficiency (60–90%)^106^ and spatial resolution (compared with a 65-megapixel film^148^), which degrade the spectral imaging performance. Besides, SLM usually requires precise alignment and correction for different wavelengths, and the system may become bulky (compared with metasurface and DOE) and expensive (tens of thousands of dollars^105^), limiting its widespread use in integrated designs and devices. Finally, SLM’s stability and reliability are easily affected by environmental fluctuations such as mechanical vibration.

### Metasurface

Metasurface is a planar optical element comprising arrays of subwavelength meta-atoms (~100 nm thickness)^130,141^. The structure introduces a sudden change of optical properties on the surface, breaking the dependence of traditional optical elements on the propagation path. This structure can interact with the incident electromagnetic field and significantly manipulate the amplitude, phase, and polarization, enabling indirect, compact, and efficient spectral modulation. By nanofabrication, it exhibits comparable modulation properties to those of conventional optical elements such as diffraction gratings and filters^109,149^, which provides a novel and compact alternative for spectral modulation.

McClung et al. designed a parallel array of metasystems for spectral modulation and imaging^149^. The array consists of metasurface-tuned filters and a metalens doublet, mapping the narrowband spatio-spectral information into different detection units. The 20 mg compact device realized multispectral imaging of ~58,000 pixels and 20 channels in the 795–980 nm range. Makarenko et al. exploited end-to-end training to design an optimized metasurface layer structure, and achieved multispectral imaging with 512 × 512 pixels from 400 to 1000 nm (204 bands)^150^. Zhang et al. established an end-to-end neural network for the joint optimization of metasurface structure and image decoding. This approach leverages the strong dispersion of metasurface to construct a point spread function that varies from 460 nm to 700 nm (25 channels), improving 800 × 800 image’s reconstruction accuracy with up to 1 dB^151^.

Despite the above demonstrations, metasurface faces several limitations. The fabrication of metasurface requires ultra-high precision and costly nanofabrication, which limits its scalability and increases production cost^116^. In addition, the conversion efficiency of metasurface for different wavelengths is different, leading to a certain amount of light energy loss^151^.

### Others

In addition to the above widely adopted spectral modulation elements, there are some other alternatives for spectral modulation. For instance, Zhang et al. reported a handheld snapshot multi-spectral camera (THETA) based on a multiplexed wavelength-dependent encoding film^148^. This system realized 65-megapixel videography of 12 wavebands within the visible wavelength range, with 25 nm spectral resolution. Besides, tunable filters such as Liquid Crystal Tunable Filters (LCTFs)^152^, Acousto-Optic Tunable Filters (AOTFs)^153^, and Fabry-Perot Tunable Filters (FPTFs)^154^ have also found applications in spectral modulation^155^. For instance, Wang et al. employed LCTF to build a compressive spectral imaging camera^156^, which used an accurate transmission model to represent LCTF’s bandpass spectral filtering effects, with a randomly coded aperture placed behind it to modulate spectra in the spatial domain. With computational reconstruction, the camera overcame the intrinsic low spatial resolution limitation of traditional LCTF spectral systems (approximately 50 × 50 pixels^157^) and achieved high-resolution multispectral imaging of 400 × 400 pixels in 500–710 nm with 10 nm spectral resolution. Although tunable filters are becoming increasingly smaller and more precise^158^, their multiple acquisitions degrade imaging speed and limit their application. In most recent years, new tunable devices featuring micro-nano structures and dynamic filtering capabilities have emerged^159^, which hold great potential for further applications in computational spectral imaging with end-to-end optimization.

## Conclusion and discussion

This review lies in the joint encoding-decoding optimization framework for computational optics and discusses a series of modulation elements for physical twinning that implements optimized parameters regarding different modulation dimensions of amplitude, phase, and spectrum. The concept of combining physical models with mathematical computation aligns with similar ideas in the fields of unrolling network^160^ and physics-inspired network^161^, underscoring that interdisciplinary study is becoming a powerful technique to tackle various challenges. With the advancement of artificial intelligence, materials manufacturing, and physics modeling, end-to-end jointly optimized computational optics will assist in high-efficiency and reliable tasks in various fields including visual reality, remote sensing, biomedicine, *etc*.

In Table 1, we briefly outline the advantages and disadvantages of the representative modulation elements discussed in each modulation dimension, aiming to guide readers in selecting appropriate modulation devices for their specific task requirements. To better illustrate their features, we also list common performance parameters in Table 2. Furthermore, as shown in Table 3, we summarize their representative applications and include a column indicating whether each device can be adaptively designed. If so, these flexible devices can be applied for adaptive modulation tasks. In contrast, the modulation devices of fixed structures are less suitable for tasks requiring adaptive modulation. The table also shows that some devices, such as DMD and LED, are commonly limited to amplitude-dimensional imaging applications. In contrast, the devices such as SLM, DOE, and metasurfaces demonstrate powerful applications in both the phase and spectral dimensions.

**Table 1:** Comparison of different modulation elements in the dimensions of amplitude, phase, and spectrum

Modulation dimension	Modulation element	Advantages	Disadvantages
Amplitude	Deformable Micromirror Device (DMD)^19,22,44,46,50,51,54–60,167^	High spatial resolution, high-speed modulation, high reliability	Complex optical systems, high cost, limited grayscale modulation performance
	Liquid Crystal (LC)^63,105^	Low cost, easy to integrate	Slow response speed
	Light-Emitting Diode (LED)^74,75,168^	Good color performance, high-speed modulation	Limited spatial resolution, high-temperature sensitivity
Phase	Diffractive Optical Element (DOE)^92,169,170^	Low cost	Large scale
	Phase-only SLM^103^	High adaptability, flexible application	High cost, complex calibration
	Metasurface^30,171,172^	Highly customizable, high precision, ultra-thin design	High cost, sensitivity to environmental conditions
Sprectrum	Filter array^136,137^	High spectral resolution, high flexibility	Finite bandwidth, low flexibility
	Diffractive Optical Element (DOE)^34,142,143^	Low cost, high throughput	Sensitivity to environmental conditions
	Spatial Light Modulator (SLM)^147,173,174^	High-speed modulation, high flexibility	Sensitivity to environmental conditions, high cost
	Metasurface^116,130,141,149–151^	Compact structure, efficient modulation	High cost, limited scalability

**Table 2:** Modulation performance of representative modulation elements

Modulation element	Model	Spatial resolution (pixels)	Modulation speed	Bit-depth / Phase modulation range	Efficiency	Spectral range
DMD	Texas DLP230NP	1920 × 1080	100 kHz (1 bit)	1–8 bit	–	400–700 nm
	Texas DLP9500UV	1920 × 1080	> 23 kHz (1 bit binary) and > 1700 Hz (8 bit gray)	1–8 bit	94% (fill factor)	363 to 420 nm
LC	Seacomp 128240C	240 × 128	64 Hz	1–24 bit	–	–
	KSXGA 0.88" M249 FLCOS	1280 × 1024	3.2 kHz for binary pattern and 85Hz for 24 bit video	1–24 bit	> 96% (fill factor)	430–700 nm
LED	LDM788AX (Xu et al.^75^)	32 × 32	1 kHz for binary pattern	8 bit	–	613–630 nm
	Salvador-Balaguer et al.^76^	32 × 32	40 kHz for binary pattern	8 bit	–	–
DOE	Beam shaper	1–10 μm	real time	0–2π	80–90%	–
	Grating	600–1800 lines/mm	real time	0–2π	30–60%	400–1000 nm
Filter array	Multispectral Filter Arrays^135^	512 × 512	real time	–	-	469–633 nm
	Broadband Encoding Stochastic (BEST) filters^136^	480 × 640	real time	–	16 filters, 40–60% fewer than required in previous studies	400–700 nm (1 nm)
Metasurface	Diffraction-based metasurface^151^	800 × 800	real time	0–2π	~ 40%–80%	795–980 nm
	Metasurface projector^149^	~ 58,000	real time	0–2π	–	400–700 nm
Phase-only SLM	HOLOEYE PLUTO-2.1 SLM	1920 × 1080	60 Hz	0–2π	87% (fill factor)	420–850 nm
	Meadowlark Optics LCoS SLM	1920 × 1152	60 Hz	0–2π	93% (fill factor)	450–1550 nm

**Table 3:** Representative applications of different modulation elements in the end-to-end joint optimization framework

Modulation element	Representative applications	Dynamic adjustment
Deformable Micromirror Device (DMD)	Single-pixel imaging and sensing^19,22,44,45,50,52–54,56–58,58,59,61,62^, optical neural network^60^, terahertz imaging^46^, infrared imaging^48,49^	✗
Liquid Crystal (LC)	Structured Illumination Microscopy (SIM)^69^, lensless single-pixel imaging system^63^, D^2^NN^103^, hyperspectral/ multispectral imaging^175^	✗
Light-Emitting Diode (LED)	Single-pixel imaging^75^, 3D holography^76^, snapshot hyperspectral imaging^173,176^	✗
Diffractive Optical Element (DOE)	Image deblurring^92^, 3D holography^93,177^, hyperspectral/multispectral imaging^139,142,143^	✗
Metasurface	Holography^110–113^, D^2^NN^30,114,115^, hyperspectral/multispectral imaging^149–151^	✗
Filter array	Hyperspectral/multispectral imaging^134,135,137,178,179^	✗

Despite the above successful demonstrations, end-to-end jointly optimized computational optics and corresponding physical twinning still face several inherent challenges that necessitate further investigations.

- *A universal framework for end-to-end optimization*
  The myriad imaging and sensing tasks exhibit various requirements in terms of computation time, network scale, decoding precision, and so on. Consequently, a delicate balance should be carefully considered between encoding efficiency and decoding quality, mandating distinct network structures for each task alongside comprehensive and repeated network learning stages. This leads to high computational complexity in the training stage, which presents an impediment to the widespread adoption of end-to-end joint optimization, particularly in scenarios with limited computational capabilities. To address this challenge, for instance, sampling-adaptive approaches^59,162^ focus on identifying common parameters while prioritizing adjustments solely to the sampling ratio, thereby minimizing alterations to a small subset of network parameters. We advocate for a deeper exploration of shared characteristics among diverse tasks and the development of a unified architecture that considers these common parameters. Such an approach would alleviate the computational burden associated with end-to-end joint optimization, thereby facilitating its broader implementation.
- *Information security*
  Within the framework of end-to-end optimization, physical twinning represents the reverse implementation of the optimized encoding parameters. The modulation devices, illumination matrices, and other information related to this stage can be considered key parameters of the neural network layers, encompassing substantial inference information about the target^163^. For certain applications such as medical diagnostics and remote sensing, information leakage can lead to significant and unexpected privacy issues. Therefore, in end-to-end optimized computational optics tasks, it is essential to raise significant concerns about protecting both the decoding network model and hardware information used in physical twinning.
- *Multi-dimensional joint optimization*
  Although existing studies have demonstrated multi-dimensional joint imaging such as holographic video imaging^164^ and hyperspectral video imaging^165^, most of them neglect the high interrelationships among different dimensions. This encoding strategy not only leads to a substantial increase in the amount of collected data but also results in notably high computational complexity and low efficiency in subsequent decoding operations. We advocate for further research efforts aimed at end-to-end joint optimization and global data compression across multiple dimensions. By utilizing a DNN structure for encoding simulations rather than relying on a single network layer or operation, discrete single-dimensional modulation elements could potentially be jointly optimized for multidimensional modulation, enhancing overall efficiency and performance.
- *Modulation element development*
  Despite the various modulation elements introduced above, their performance is still not enough to afford the rising requirements of various applications. To better match the high-precision parameters optimized by neural networks, there is an increasing demand for delicate modulation elements with higher modulation resolution, bit depth, modulation efficiency, and stronger stability. In addition, as optical systems advance towards miniaturization and integration^166^, micro-nano fabrication technologies and the development of intelligent multi-dimensional modulation elements will emerge as key focuses and challenges in the field of computational optics.

## Data Availability

Data underlying the results presented in this paper are not publicly available at this time but may be obtained from the authors upon reasonable request.
